# Video Summarization Based on Mutual Information and Entropy Sliding Window Method

**DOI:** 10.3390/e22111285

**Published:** 2020-11-12

**Authors:** WenLin Li, DeYu Qi, ChangJian Zhang, Jing Guo, JiaJun Yao

**Affiliations:** 1 School of Computer Science and Engineering, South China University of Technology, Guangzhou 510006, China; zhangcj.work@gmail.com; 2 School of Software Engineering, South China University of Technology, Guangzhou 510006, China; guojing16.cs@gmail.com (J.G.); yaojiajun131@gmail.com (J.Y.)

**Keywords:** entropy, video summarization, key frame extraction, video analysis, gesture videos, feature extraction

## Abstract

This paper proposes a video summarization algorithm called the Mutual Information and Entropy based adaptive Sliding Window (MIESW) method, which is specifically for the static summary of gesture videos. Considering that gesture videos usually have uncertain transition postures and unclear movement boundaries or inexplicable frames, we propose a three-step method where the first step involves browsing a video, the second step applies the MIESW method to select candidate key frames, and the third step removes most redundant key frames. In detail, the first step is to convert the video into a sequence of frames and adjust the size of the frames. In the second step, a key frame extraction algorithm named MIESW is executed. The inter-frame mutual information value is used as a metric to adaptively adjust the size of the sliding window to group similar content of the video. Then, based on the entropy value of the frame and the average mutual information value of the frame group, the threshold method is applied to optimize the grouping, and the key frames are extracted. In the third step, speeded up robust features (SURF) analysis is performed to eliminate redundant frames in these candidate key frames. The calculation of Precision, Recall, and F${}_{measure}$ are optimized from the perspective of practicality and feasibility. Experiments demonstrate that key frames extracted using our method provide high-quality video summaries and basically cover the main content of the gesture video.

## 1. Introduction

Nowadays, camera-based human-computer interaction (HCI) is increasingly being applied to intelligent life. The technological advancements in video acquisition approaches led to independence in video storage capacities [1]. Extracting the frames that can express the main content of video sequences, called key frame extraction, is one of the important methods of video summarization [2]. Due to the diversity features of different videos, there is no universal key frame extraction algorithm, especially for specific content. As a more convenient way of communication, gesture video analysis is widely studied in the field of human-computer interaction. Effectively extracting key-frames from gesture video repositories is still a challenging task. According to the structure information and diversity features, there are four major categories of methods used in key frame extraction.

The first method is based on shot detection and considers key frame extraction as a shot segmentation problem. In [3], the key frames are extracted by using histogram features based on the shots change detecting. This method is suitable for video sequences with less shot changes and simple content with fewer redundant frames.

The second major category of key frame extraction is based on clustering, where frames are clustered into groups according to the principle of similarity. In [4], a modified k-means clustering method is used to extract key frames. In [5], video summarization is formulated as a sequential decision, and treated diversity reward measure as a k-medoids problem; therefore, cluster centers are selected as key frames. The clustering algorithm is an unsupervised learning process and it is sensitive to dirty data. It has a large amount of computation, which is more suitable for small data calculation.

The third type of method is based on motion features such as motion vector estimation [6] and the optical flow method [7]. However, the motion feature is usually local information, and for locally complicated videos, using a motion estimation method may extract many redundant frames. Optical flow estimates the displacement area between two frames, providing motion and structure information. Because the calculation is the displacement of sub-pixel, there will be higher accuracy for motion estimation and more suitable for complex movement; however, pixel-level processing may result in excessive computation.

The fourth method is based on feature extraction and analysis, which uses feature descriptors such as color characteristics and texture as the basis for analysis. By setting appropriate threshold parameters, key frames with significant variations are extracted through inter-frame feature comparison [8].

In general, video retrieval technology includes: scene analysis, video structure processing, shot segmentation, sequence feature extraction, video content summary, and so forth. Above all, key frame extraction is the premise of indexing and summarization for the video sequence. These algorithms either use certain features to distinguish key frames, resulting in inaccurate extraction when the features are insufficient or disturbed by noise, or based on pixel-level motion extraction, which may lead to excessive calculations. To address these problems, we propose a key frame extraction method for gesture video called the Mutual Information and Entropy based adaptive Sliding Window (MIESW). The first step is to preprocess the video into a series of frames and resize the image. The second step refers to a rough temporal segment—it divides pre-processed videos into short segments by employing an improved sliding window with mutual information and entropy value as metrics for the sake of extracting candidate key-frames. In the third step, we apply speeded up robust features (SURF) features to determine similarity between candidate key-frames, then remove frames with high similarity to eliminate redundancy of key-frames sequences. As a result, the most representative frames can basically cover the main content of the given video. Precision, recall, and F-measure criterion are optimized conceptually, aiming to more accurately evaluate the pros and cons of the algorithm.

The remaining chapters of the paper are arranged as follows: Section 2 covers related work, Section 3 introduces algorithms and experimental methods, Section 4 conducts the experiment and clarifies results, and Section 5 summarizes the content of the paper and points out future work.

## 2. Related Work

Indexing, matching, and summarization of video sequences are popular areas, and the most popular method is based on key frame extraction. In order to extract key frames, domestic and foreign experts have conducted extensive research in different fields.

Shot detection are methods of extracting key frames. Rachida et al. [9] proposed a technology based on shot clip detection and video summarization, and used it in the editing of movies, documentaries, sports, and other videos. This method uses adaptive mean shift algorithm and global orientation features to build a key frames extraction method termed mean shift-based keyframes for video summarization (MSKVS). The algorithm proposed global frame feature vector (GFFV) descriptor and used it to describe visual content. However, the mean shift algorithm may get stuck at a saddle point, and more analysis should be done to make MSKVS suitable for real-time scenarios. In the action shot summary method proposed by Meghdadi et al. [10], the summary of the action shot is completed by merging still images aligned with the action frame. The algorithm relies on the motion trajectory of the action to generate the shot summary. When there are multiple targets or occlusions in the video, this may cause segmentation errors of the action shots. In the field of visual positioning, key frames extraction approach based on shot detection is used to construct an offline image database to store visual maps with location information. The algorithm proposed by [11] presets the first key frames and uses fixed-size groupings as shots to select key frames instead of grouping according to the similarity of content, which may lead to a decrease in the accuracy of key frame extraction.

The cluster-based key frames extraction methods are also very popular. In [4], a method based on sparse coding and k-means clustering is used to extract key frames, and added some conditions to select the number of key frames from each shot. However, these conditions are relatively harsh, and the constraint conditions of the sparse coding vector quantization method are too strict, which leads to more complicated parameters for extracting key frames. Avila et al. [12] propose a method of static video summaries based on color feature and K-means clustering. In [5], video summarization is formulated as an unsupervised decision process, and treated diversity reward measure as a k-medoids problem. Since it is necessary to specify the number of cluster centers, that is, the number of key frames obtained, this may result in key frame missed detection or excessive detection due to incorrect parameter settings. There are also some improved algorithms for extracting key frames based on semantic clustering. For example, Yin et al. [13] constructed an innovative algorithm based on semantic connections among the elements. In order to construct SeTree, a normalized graph cut clustering algorithm by combining visual features, textual information and user preferences is proposed. For key frames extraction, apart from using clustering to detect shot boundaries, it also uses personalized saliency and spatio–temporal saliency to score the importance of segments. Nevertheless, this algorithm needs to encode and cluster image features, which is slightly complicated for occasions with high real-time requirements. Generally, the clustering algorithms used to extract key frames are unsupervised learning processes that require clustering centers and are sensitive to dirty data. The clustering methods may also have the disadvantage that the obtained key frames lose the time information of the original video. These method usually have large amounts of calculation and are more suitable for small data calculations.

The key frames methods based on motion feature extraction have also attracted people’s attention. Zhu et al. [14] maps the original high-dimensional data to the low-dimensional state space, clusters motion data, and uses the segmentation points as the extraction basis of key frames. However, this method adopts the idea of dimensionality reduction, combined with motion features, it will lose local features and may lead to incorrect results. Wolf [15] first proposed the method of using optical flow calculation to extract key frames. Inspired by Wolf, Gao et al. [16] proposes a method of summarizing video using optical flow tensor (OFT) combined with hidden Markov model (HMM). The combination of the two methods can accurately capture the motion information of the video, but this method is sensitive to the video content and is more suitable for precise video extraction of subtle differences. Optical flow estimation is a pixel-level calculation method, which estimates the displacement by calculating the gray change value in two frames. Using optical flow estimation algorithms to extract key frames require that the brightness and space of the video remain almost constant, which greatly limits the application of the algorithm.

Scholars have also done some research on constructing feature descriptors to extract key frames from videos. Extraction algorithms based on image features are usually all non-domain specific and general. In [17], color features and structural features are used to describe the feature of the image content. In [18], the local feature SURF is selected to extract local points as the frame feature descriptor, and then used to analyze the frame sequence with the sliding window and extract key frames. In each shot, the most typical frames are extracted as [19] calculates the characteristics of the energy and standard deviation of each sub-band after contour wave transformation and forms a feature vector representation to extract key frames. The method of extracting key frames based on a single feature cannot effectively express the video content to some extent, especially when the video shots is complicated.

The above research progress on key frames is mainly for specific areas such as medicine, animation, content extraction, indoor visual positioning, monitoring analysis, human motion data capture, etc. In these fields, key frame extraction technology provides convenience for quickly and accurately understanding relevant video content. Gesture recognition is a prevalent way of human-computer interaction. Research on dynamic gesture recognition can better optimize the real-time requirements of human-computer interaction. However, dynamic gesture videos generally contain hundreds of frames, which complicates the task of directly analyzing and processing video data. By using key frame extraction technology to summarize the main content of the video, the calculation of redundant information can be reduced and the real-time performance of human-computer interaction can be improved; therefore, it is of great significance to extract key frames from gesture video as an advanced gesture recognition algorithm.

## 3. Key Frames Extraction and Feature Fusion Principle

The key frame extraction method proposed in this paper is mainly divided into the following steps, namely, extracting image information, optimizing image information construction efficiency to improve storage occupation, and analyzing the relationship between video sequences to extract representative frames. The principles, feasibility and experimental demonstration of each step are separately described below.

### 3.1. Entropy and Mutual Information Theory

As the basis of information theory, since 1995, mutual information has been applied to the fields of image comparison and multi-modal medical image matching [20] and has gradually been applied to shots detection [21] and image segmentation. The literature [22] combines the mutual information algorithm and k-means method to segment medical and automobile images. For a given image, it assumes that pixel gray values are independent and appear randomly in the image. The image distribution is expressed as $P=\{p_{0},p_{1},\ldots p_{L-1}\}$, where $p_{i}$ represents the image and the gray value.

For the image frames *X*, their entropy H[X] can be expressed as (1):

(1) $$\begin{array}{c} H\left[X\right]=-\sum_{k}p_{X}\left(k\right)\ln\left(p_{X}\left(k\right)\right) \end{array}$$

where $p_{X}\left(k\right)$ denotes the probability density function of frames *X*, the probability function can be obtained by normalized histogram of the gray-scale pixel.

Mutual information between *X* and *Y* noted as I[Y,X], which can be defined as the difference between entropy of *X* and conditional entropy of *Y* given the condition of *X* noted as H(Y|X) as follows:

(2) $$\begin{array}{ccc} I[Y,X] & = & H[X]-H[X|Y] \end{array}$$

In this paper, X,Y denote frame images, then the joint probability distribution is the normalized histogram of the two images. Mutual information is applied in video extraction as a basis for judging the similarity between frames. If the mutual information value is greater than the threshold, that means the content of the two frames has changed a lot. Entropy H[X] measures the degree of change in gesture movements. The H[X,Y] indicates the similarity of the two frames.

**Figure 1:**
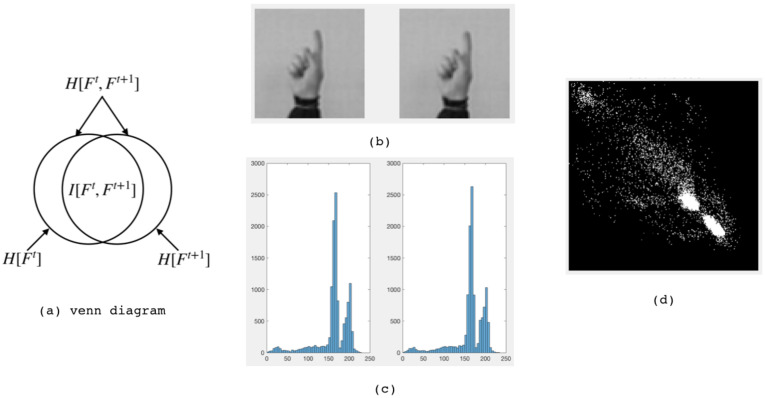
Entropy H[F] measures the degree of change in gesture movements. The correlation entropy $H[F^{t},F^{\left(t+1\right)}]$ indicates the similarity of the two frames. The mathematical relationship between information entropy and mutual information is illustrated in (**a**), $F^{t}$ denotes a frame at time *t*, and $H[F^{t}]$ represents the information entropy of $F^{t}$, $I[F^{t},F^{t+1}]$ represents the mutual information value of two consecutive frames at time *t* and t+1. (**b**) Shows two gray-scale gesture images, and (**c**) shows the gray-scale distribution histograms, which can be used to calculate the entropy of the image. In (**d**), the horizontal axis represents the gray gradation value, and the vertical axis shows the number of pixels. The joint histogram counts the frequencies at which different gray-value combinations appear at corresponding positions in the two images. The shapes of the two histograms are similar, indicating that their probability distribution of the pixel gray values in approximate.

In Figure 1, Venn diagram represents the relationship between image entropy, joint distribution and mutual information. The histogram represents the probability distribution of grayscale images.

The key frame extraction algorithm based on entropy and mutual information involves the following steps:

1. Input video: Input the video to extract key frames.
2. Conversion part: Convert the video into a frame sequence, count the length of the sequence, and adjust the frame size to N×N.
3. First, an adaptive sliding window is used to process the frame sequence. The mutual information value is calculated for the frame group entering the window, the window size is adaptively adjusted according to the difference between the frames, and similar frames are classified into a group. Then according to the entropy value of the frame, the groups with similar content are further merged, and finally the frame closest to the average mutual information value between the groups is selected to enter the candidate sequence of the key frame.
4. Eliminate redundancy: Use SURF to extract the features of the frames in the candidate sequence, and eliminate the redundant frames with high similarity.
5. Output key frames: Output the final key frame extraction results. It should be noted that during the key frame extraction process, the frame sequence retains time sequential information.

**Figure 2:**
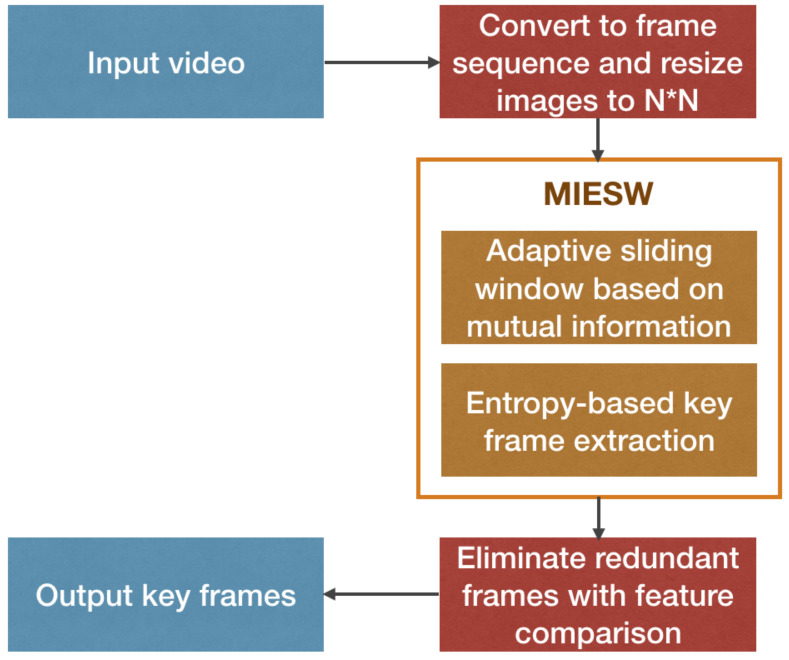
Flow chart of the Mutual Information and Entropy based adaptive Sliding Window (MIESW) algorithm for extracting key frames.

Figure 2 elaborates the mechanism used in the proposed solution. The input and output modules are represented by blue squares, the red squares represent the pre-conversion module and the candidate sequence adjustment module, and the inside of the yellow square is the MIESW algorithm module.

### 3.2. The Proposed Mutual Information and Entropy Based Sliding Window Method (MIESW) for Key Frames Extraction

The key frame extraction method proposed in this paper is called MIESW, which is mainly divided into the following steps: calculate the inter-frame mutual information value to acquire spatial-temporal information, use an adaptive sliding window method to group similar content frames, and apply an entropy-based threshold method optimization grouping. As a result, a candidate key frame sequence is obtained. In order to obtain the key frame sequence, the feature extraction algorithm is used to analyze the similarity of the frames in the candidate frame sequence and eliminate redundant frames.

### 3.3. Improved Adaptive Sliding Window Method to Extract Key Frames

Traditional key frame extraction algorithms based on sliding window measure the similarities between adjacent frames. The purpose of processing data using a sliding window is to analyze the local features and overall characteristics of the video sequences. In [23], combined with ensemble learning, a sliding window-based support vector regression method is proposed to predict micrometeorological data. In [24], three consecutive sliding windows are considered to calculate G1-continuities errors as the criterion. This method takes the shots boundaries and transitions types into consideration, however, calculation may be huge. The fixed window size will also result in poor adaptability of the sliding window algorithm to different types of video sequences. This method considers the boundary and transition type of the camera, but the amount of calculation may be large.

**Figure 3:**
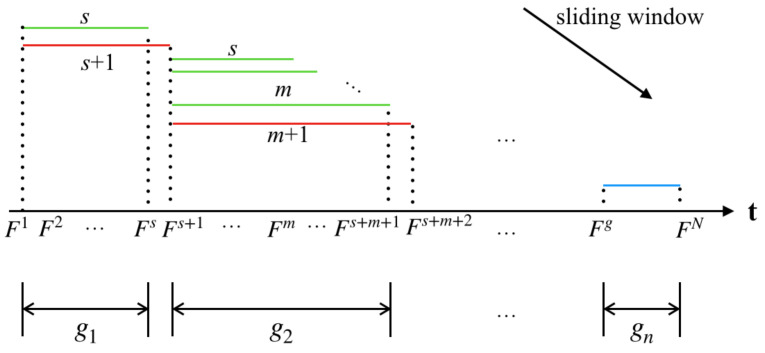
The improved sliding window schematic.

Taking these factors into consideration, an improved the sliding window method is proposed, which can be seen in Figure 3. In the segmentation algorithm proposed in paper, we calculate the average similarity on mutual information between the frame we have traveled and frames within the current sliding window.

**Figure 4:**
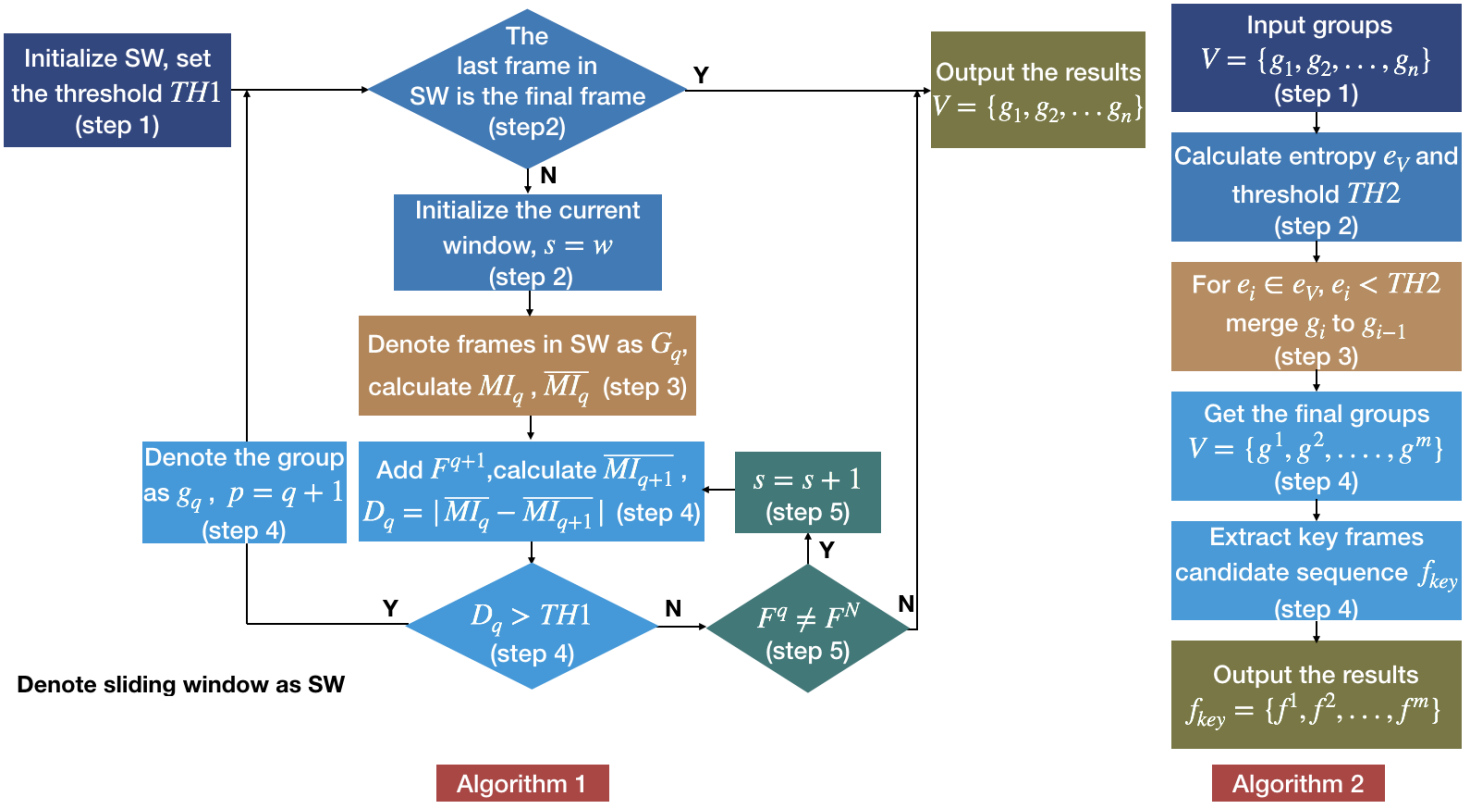
The framework of Algorithms 1 and 2.

As shown in Figure 3, $F^{1}$ represents the first frame in the video sequence *V*, and $F^{N}$ indicates that the sequence has *N* frames. The *t* axis represents a time-line. In Figure 3 the green line represents the sliding window, the changing in its length represents the process of adaptively changing the size of the sliding window, and the red line represents the size of the window no longer increases. The frames in the fixed window are regarded as a group. The last group is represented by a blue line, because the length of the frame sequence entering the sliding window may be less than the initial value of the sliding window. In this case, the frame sequence is directly grouped and marked as $g_{n}$. The grouped frame sequence is denoted as $V=\{g_{1},g_{2},\ldots ,g_{n}\}$. The key frames extraction framework named MIESW consists of two stages: segmentation and grouping, which are summarized as Algorithms 1 and 2. The framework of Algorithms 1 and 2 are shown in Figure 4.

We designed Algorithm 1 as follows: When using the MIESW method to extract key frames, we use the adaptive sliding window method to group the frame sequence with primary similarity according to Algorithm 1. First, initialize the sliding window, set the threshold (step 1), then determine whether the last frame that entered the sliding window is the final frame (step 2). Next, we calculate mutual information value of the frame group in the sliding window (step 3), and then add the frame next to the current window’s right boundary to judge the degree of similarity changes within the group (step 4). Thereafter, the size of the sliding window is adaptively adjusted according to the threshold (step 5), and the frame group are output after the loop ends. The detailed algorithm is as follows:

step 1. Pre-defined the initial length of the sliding window to *w*. Set the threshold TH1. Start the algorithm from the first frame.
step 2. Make sure the last frame in the sliding window is not the final frame $F^{N}$. If true, denote the current group as $g_{n}$, algorithm is over, output the result. Otherwise, initial the current window size s=w, go to step 3.
step 3. The window slides on the sequence, and frames entering the sliding window are denoted as $G_{q}=\left\{F^{p},F^{p+1},\ldots ,F^{q}\right\}$, satisfying: q=p+s. Calculate the mutual information value M of each consecutive two frames in $G_{q}$, recorded as $MI_{q}=\left\{M_{p},M_{p+1},\ldots ,M_{q}\right\}$, and then calculate the mean value as $\bar{MI_{q}}=\sum_{i=p}^{q}MI_{pq}/s.$
step 4. Add the frame next to the current window’s right boundary $F^{q+1}$ into the group and obtain $\bar{MI_{q+1}}$. Calculate the absolute value of the difference between $\bar{MI_{q}}$ and $\bar{MI_{q+1}}$ as $D_{q}$. $D_{q}=\left|\bar{MI_{q}}-\bar{MI_{q+1}}\right|$ If $D_{q}>TH1$, it means that the newly added frame $F^{s+1}$ reduces the overall correlation of the original group $G_{q}$, so denote the group as $g_{q}$. Set p=q+1, go to step 2. Otherwise go to step 5.
step 5. $D_{p}<TH1$, it indicates that the newly added frame has a correlation with the original group. If $F^{q}\neq F^{N}$, then s=s+1, go to step 4. If $F^{q}=F^{N}$, go to step 2.

After the first grouping, the video sequence is approximately divided into some groups. Moreover, to merge similar adjacent groups, we perform Algorithm 2. Algorithm 2 is used to extract candidate key frames. Use the frame groups output by Algorithm 1 as input (step 1), then calculate the entropy indicator and threshold (step 2). After high similarity groups are merged according to the entropy indicator and the threshold (step 3), candidate key frames are extracted from the new groups based on mutual information characteristics (step 4). The detailed algorithm is as follows:

step 1. Represented groups in the sequence as $V=\{g_{1},g_{2},\ldots ,g_{n}\}$ as input.
step 2. Calculate the standard deviation of the entropy values of each group as $e_{V}=\left\{e_{1},e_{2},\ldots ,e_{n}\right\}$, and the average standard deviation is obtained as the threshold $TH2=\sum_{1}^{n}e/n$.
step 3. In the second grouping, adjacent segment which is smaller than the threshold TH2 will be merged. For $e_{i}\in e_{V}$, if $e_{i}<TH2$, merge $g_{i}$ to $g_{i-1}$; if not, keep the group unchanged.
step 4. After this process, the final groups is denoted as $V=\{g^{1},g^{2},\ldots .,g^{m}\}$, in each group, the frame closets to the average mutual information value is selected as the key frame: $f_{key}=\left\{f^{1},f^{2},\ldots ,f^{m}\right\}$.

**Figure 5:**
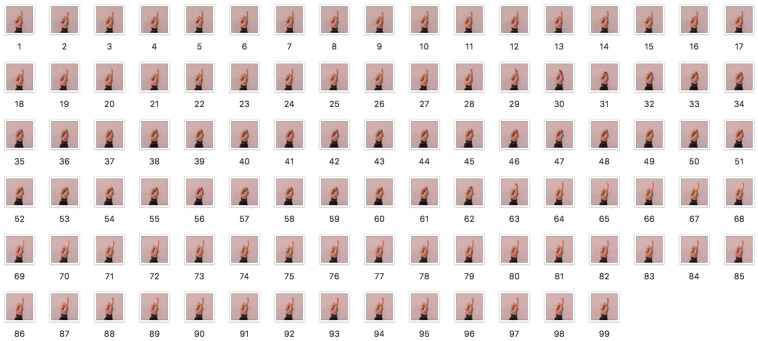
Frames in the video of a gesture for the word `Clic’.

**Figure 6:**
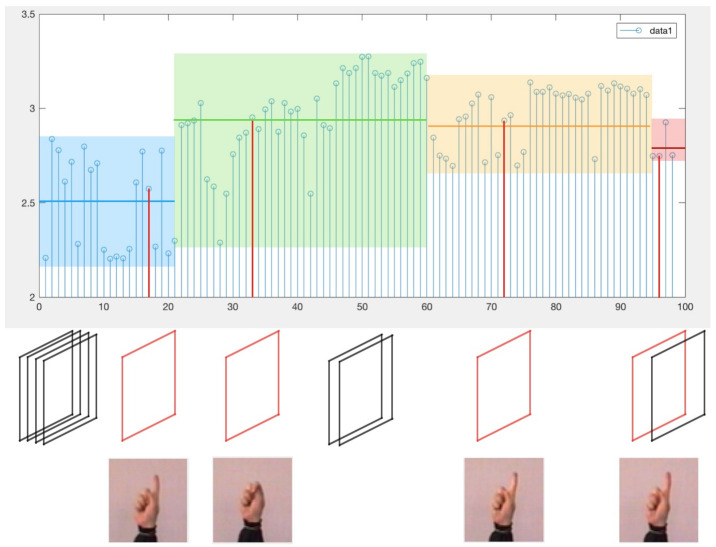
Schematic diagram of extracting key frames. A hand gesture sequence sample from Marcel-IDIAP2001, which contains 99 frames. The horizontal axis coordinate represents the mutual information value obtained by successive frame pairs in the video sequence. Data1 is the inter-frame mutual information value range from 2 to 3.5. Blocks of different colors indicate the grouping results after using Algorithms 2 and 3. The horizontal lines in color blocks indicate the mean value of each group. The key frames obtained by our method are in red boxes, which are the 17, 33, 72, and 98 frames.

Figure 5 shows a sequence of frames from the gesture sequence `Clic’, which contains 99 frames. The detailed process of the MIESW algorithm is shown in the Figure 6, the green line indicates the number of frames included in the frame segment which is the frame group length. The red line indicates that the newly added frame does not meet the group condition. For the first frame segment $g_{1}$, its length is *s*. For the case where the difference D1 is smaller than the threshold, as shown by the $F^{s+1}$ frame in the Figure 6. The segment $g_{2}$ shown in the Figure 6 has a length of m. It should be noted that if the length of the last group is smaller than the original window size *s*, the last segment is directly set to $g_{n}$ as shown by the blue line.

### 3.4. Remove Redundant Frames

To remove redundant frames from candidate key frames, we analyze sequences similarity through image feature extraction. In image processing, feature extraction has become a popular method in image classification [25], image retrieval [26], target detection [27], and image segmentation [28]. In recent years, most of the research has focused on the area of deep learning, which extracts eigenvector or characteristic vector by using different convolution kernels.

However, traditional methods with mathematical operators such as Harris Corner Detector, SURF, scale-invariant feature transform (SIFT), etc. still have the advantages in key-points orientation description, scale analysis, and illumination variation. Proposed by David, the SIFT [29] detector can find feature points on different scale spaces. As a robust local descriptor, SURF is usually used in key-frame extraction tasks. We evaluate SURF detectors as a metric to counting the number of features in each key-frame. The ultimate matching of SURF feature is to measure the similarity of the two key-frames and serve as the basis for removing redundant frames. SURF is inspired by SIFT descriptor, which is several times faster than SIFT at the cost of fewer feature points to be detected. Based on Haar wavelet transform, SURF performs Hessian algorithm to detect key points.

SURF uses a Hessian Matrix to extract feature points. The Hessian Matrix is a square matrix composed of the second-order partial derivative, which describes the local curvature of the multivariate function. For an image I(x,y), we denote the Hessian Matrix as:

(3) $$\begin{array}{c} H\left(x,y,\sigma \right)=\left[\begin{array}{cc} L_{xx}\left(x,y,\sigma \right) & L_{xy}\left(x,y,\sigma \right)\\ L_{xy}\left(x,y,\sigma \right) & L_{yy}\left(x,y,\sigma \right) \end{array}\right] \end{array}$$

with,

(4) $$\begin{array}{c} L(x,y,\sigma )=G(\sigma )*I(x,y) \end{array}$$

(5) $$\begin{array}{c} G\left(\sigma \right)=\frac{1}{2\pi \sigma^{2}}e^{-\frac{x^{2}+y^{2}}{2\sigma^{2}}} \end{array}$$

**Figure 7:**
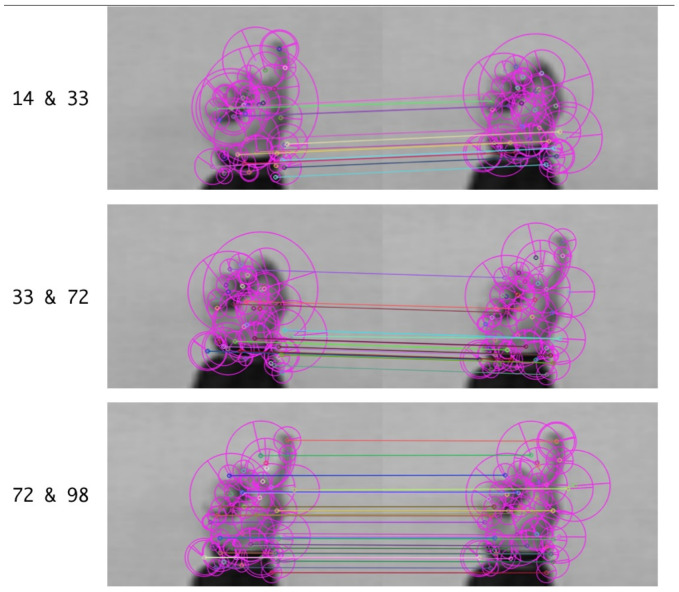
SURF (Speeded Up Robust Features) analysis.

SURF compares each pixel point processed by the Hessian matrix with its image domain (image of the same size) and all neighboring points in the adjacent scale space. When it is greater than (or less than) all neighboring points, the point is feature point. In the SURF algorithm, it counts the Harr wavelet features in the circular neighborhood of the feature point with the fan-shaped area as a unit, and takes the fan-shaped direction of the largest feature value as the main direction of the feature point. SURF has excellent scale-invariant features which are advantageous for specific target-gesture feature extraction and image comparison. SURF vector matching is used for the candidate frame sequences to eliminate redundant frames. Taking the experimental sequence as an example, the candidate key-frames numbers are 14, 33, 72, and 98. We evaluated the SURF detectors ability to process matching images—the results are shown in Figure 7:

Analyzed from the Figure 7 and Table 1, the number of point pairs on SURF matching in the third figure is the maximum compared to the first and the second figure. In other words, the frame 72 and the frame 98 have a high degree of similarity. We calculate the similarity between candidate key-frames by analyzing SURF features and matching degree.

**Figure 8:**
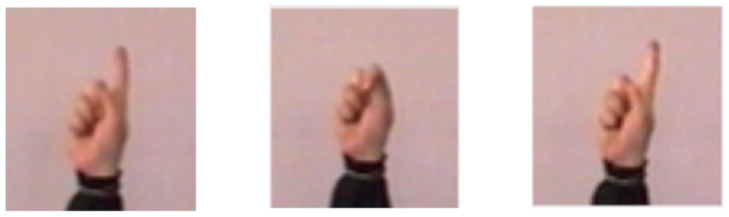
The key frames extraction result of the test video.

SURF similarity describes the resemblance of candidate frames in visual features. If the indicator is as high as 50%, it demonstrates that there are redundant frames in the candidate frames, and redundant frames will be eliminated. Based on this method, key-frames are determined. So the extraction results of this video is shown as Figure 8.

## 4. Experiment

### 4.1. Improved Precision, Recall, and F${}_{measure}$ Criteria

Three quantitative assessment metrics based on the visual comparison which have been used to evaluate the quality of keyframes extraction methods, i.e., Precision, Recall, and F${}_{measure}$, that are defined as follows:

(6) $$\begin{array}{c} P=\frac{N_{m}}{N_{AK}} \end{array}$$

(7) $$\begin{array}{c} R=\frac{N_{m}}{N_{UK}} \end{array}$$

(8) $$\begin{array}{c} F_{measure}=\frac{2\times P\times R}{P+R}=\frac{2\times N_{m}}{N_{AK}+N_{UK}} \end{array}$$

where *P* denotes Precision, *R* denotes Recall, $N_{AK}$ is the number of automatic keyframes and $N_{UK}$ is the number of user-set keyframes, $N_{m}$ as the number of matching keyframes indexes between $N_{AK}$ and $N_{UK}$.

However, the quantitative metrics rely too much on the key frames set by the user, which may cause the extracted representative key frames to be incorrectly judged due to different set frame numbers. Another method [30] takes this situation into consideration and adds the parameter of similar frames to the improved metrics to reduce the misjudgment, as shown in (9)–(11):

(9) $$\begin{array}{c} P=\frac{N_{UK}}{N_{UK}+N_{S}} \end{array}$$

(10) $$\begin{array}{c} R=\frac{N_{UK}}{N_{UK}+N_{MS}} \end{array}$$

(11) $$\begin{array}{c} F_{measure}=\frac{2\times P\times R}{P+R} \end{array}$$

where $N_{S}$ denotes the number of keyframes found similar to the user-set keyframes, $N_{MS}$ is the number of missed detection of the correct result, which is user-set keyframes.

Considering the continuity of frame sequences, it is important to reduce the subjective in this method. Instead of obtaining precision and recall by counting automatic key frames and user-set key frames as shown in (6)–(8), or calculating similar user-set key frames and missed correct key frames as shown in (9)–(11), an optimized assessment metrics has been approved. Since the gestures appearing in the video are coherent, the key frame set by the user cannot be accurate to a specific value, so setting it within a reasonable range is a feasible measure. The improved evaluation metrics are as (12)–(14):

(12) $$\begin{array}{c} P=\frac{N_{s}}{N_{g}} \end{array}$$

(13) $$\begin{array}{c} R=\frac{N_{s}}{N_{u}} \end{array}$$

(14) $$\begin{array}{c} F_{measure}=\frac{2\times P\times R}{P+R}=\frac{2\times N_{s}}{N_{g}+N_{u}} \end{array}$$

where $N_{u}$ is a collection of key frame areas set by the user, $N_{g}$ is the key frame extracted by the algorithm. $N_{s}$ is the number of matched frames, which refers to the number of frames that fall correctly in the user-set key frame area set.

Only one key frame is allowed to fall in each area. If two or more key frames extracted by the algorithm fall into the same area, the correct matching number can only count one frame; if there is no algorithm extracted frame falling within a user-set key frame area, the area is determined as the missed detection area and the correct matching number is not changed in this case.

### 4.2. Experimental Results

Experiments were conducted on many videos, and some examples of experimental results are shown below. Experiments were performed on the hand posture video sequences ’Clic’ and ’StopGraspOk’ of Dr.Sebastien Marcel-IDIAP2001. The ’Clic’ sequences contain three gestures: raising the index finger, clenching fists, and then raising the index finger.

For the comparison, four different baselines were selected: (1) entropy-based (with color histograms) [31], (2) color-based [17], (3) sliding window based (with gist and sift features) [18], and (4) our method without deduplication optimization.

In Table 2, these sequences denoted as {C1, C2} and {S1, S2}, respectively. Frames are color images saved in portable any map (PNM) format with the size of 62×58×3. Therefore, in general, the cubic spline interpolation is used to adjust the image size to 64×64×3. The proposed(w/o optimization) method and our method are performed on the experimental data using the threshold of 0.3. The other three algorithms are implemented according to the settings of corresponding original papers.

Experimental results are evaluated using the modified Precision (P), Recall (R), and F${}_{measure}$ (F) measurement. Table 2 shows the results. The entropy-based [31] method calculated the entropy value of the frame to perform key frames selection, and the color-based [17] method extract color characteristics to obtain temporal segment on the video. The sliding window based [18] extracts features of frames by using gist and sift features and then use the sliding window to extract key frames. These three methods share different cogitation on temporal segments, comparing the approval method of others in the identical dataset is a fair and convincing way.

**Figure 9:**
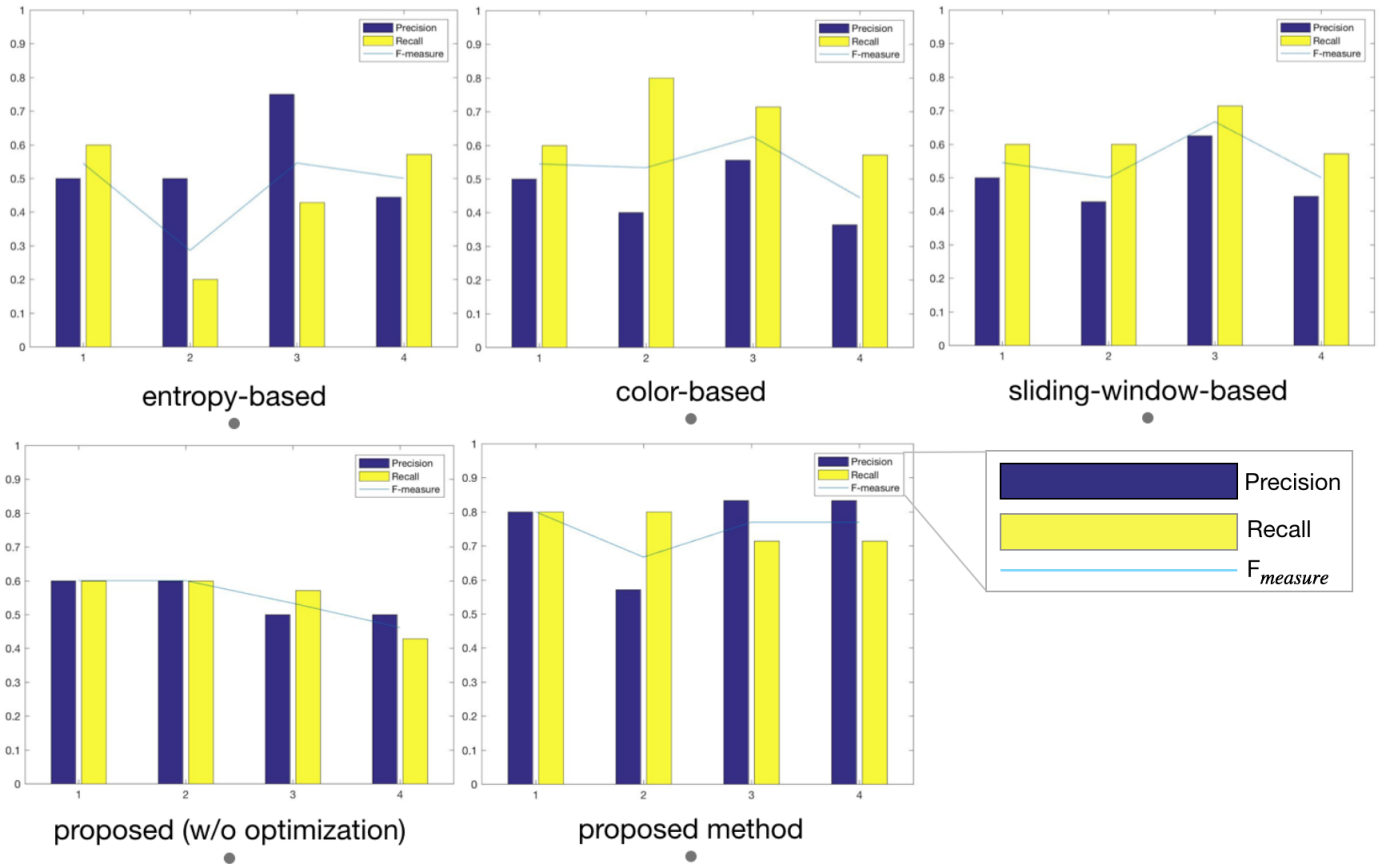
Precision, Recall, and F${}_{measure}$ of different techniques on test videos. The purple bar, yellow bar, and blue line represent Precision, Recall, and F-measure, respectively. The horizontal axis in the figure represents four test videos, and the vertical axis represents the PRF evaluation metrics of the key frame extraction result. It can be seen that using the same test video, our proposed algorithm can obtain higher precision, recall, and F ${}_{measure}$. For detailed experimental results, see Table 2.

In order to make the results more intuitive and easier for objective comparison, Figure 9 shows the PRF comparison results. The PRF criterion reflect quantitative results of key frame selection. It can be seen that our method has achieved better results. There is improvement on Precision, Recall, and F${}_{measure}$ in our algorithm compared with these algorithms.

**Figure 10:**
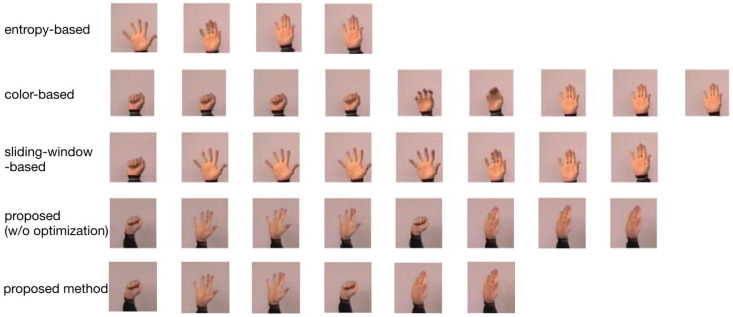
The key frames extractions applied on video S2 that contains 141 frames and consists of four gestures. Its gesture starts with the fist, then opens fingers, next clenches the fist, and finally opens the palm. For the 1st to 3rd comparison algorithms, especially the third gesture, has been leaked, which would be bad in key-frames extraction. However, in the fourth algorithm we proposed, although the third gesture was extracted, unfortunately, there are many redundant frames. The results of our proposed method shows a series of gesture changes can be extracted more obviously.

Some results of key frames extraction are shown in Figure 10. It can be seen with the comparison results that our proposed method conducted better key frames extraction for the dataset. We infer that it is because our algorithm fully considers the inter-frame information and the characteristics of frames, then performs adaptive sliding-window based adaptive grouping to extract candidate key frames, and finally eliminates redundant frames. Entropy-based method depends only on the maximum and minimum of the frames’ entropy, it may missing some key frames that are not at extreme points. Since the background color of our dataset is relatively close to the foreground color, so the color-based method does not perform well. Due to the shot almost unchanged, the sliding-window based method may have trouble dividing frames in groups. Although the proposed method without optimization has selected key frames that can represent video content, there are also many redundant frames. However, we can remove some redundant frames by performing the SURF similarity method. Using the key frame extraction algorithm proposed in this paper can increase feature extraction efficiency and eliminate redundant information.

## 5. Conclusions

In this paper, we have devised and performed a video summarization approach called MIESW for gesture videos. The innovations of this method are as follows: considering the correlation between frames, an improved key frame extraction algorithm based on adaptive sliding window is proposed. When sliding on the video sequences, the window size is adaptively adjusted to accommodate the characteristics of the sequences expressed by mutual information values. After the second frame grouping measured by entropy, it is ensured that similar content is correctly classified and representative frames are extracted as key frames. Extract the SURF of candidate key frames, and then analyze the similarity between them to delete redundant frames and obtain key frames. The calculation of Precision, Recall, and F${}_{measure}$ are optimized from the perspective of practicality and feasibility which improves the definition of user-set key frames to key frame intervals, taking into account the continuity of video content. Experimental results demonstrate that the proposed method performs well on the dataset. However, with the continuous expansion of the application range of gestures, the key frames extraction for videos in more complex backgrounds may be a limitation of our algorithm. In future, we plan to optimize the algorithm in a complex background, and use deep learning framework for automatic feature extraction. The key frame extraction algorithm proposed in this paper is not limited by video content and is applied to gesture extraction. However, the video content is varied and the video lengths are different. The increase in video services has also challenged video summarization and key frame extraction algorithms. In the subsequent improvements, we will further study the promotion of algorithm to the extraction of multiple videos.

## Figures and Tables

**Table 1:** Similarity based on SURF descriptor.

Compared Images	The Best Matching Value	The Worst Matching Value	SURF Similarity
(14, 33)	0.0414	0.5746	0.3469
(33, 72)	0.0313	0.5511	0.3864
(72, 98)	0.0187	0.6372	0.5882

**Table 2:** Comparison of Precision (P), Recall (R), and F${}_{measure}$ (F) of different techniques on the test videos. The best results are highlighted in bold.

Method	Video Name	$N_{u}$	$N_{g}$	$N_{s}$	P	R	F
entropy-based	c1	5	6	3	0.5000	0.6000	0.5445
	c2	5	2	1	0.5000	0.2000	0.2857
	s1	7	4	3	0.7500	0.4286	0.5455
	s2	7	9	4	0.4444	0.5714	0.5000
color-based	c1	5	6	3	0.5000	0.6000	0.5445
	c2	5	10	4	0.4000	0.8000	0.5333
	s1	7	9	5	0.5556	0.7143	0.6250
	s2	7	11	4	0.3636	0.5714	0.4444
sliding-window-based	c1	5	6	3	0.5000	0.6000	0.5445
	c2	5	7	3	0.4286	0.6000	0.5000
	s1	7	8	5	0.6250	0.7143	0.6667
	s2	7	9	4	0.4444	0.5714	0.5000
proposed (w/o optimization)	c1	5	5	3	0.6000	0.6000	0.6000
	c2	5	5	3	0.6000	0.6000	0.6000
	s1	7	8	4	0.5000	0.5714	0.5333
	s2	7	6	3	0.5000	0.4286	0.4615
**our method**	c1	**5**	**5**	**4**	**0.8000**	**0.8000**	**0.8000**
	c2	**5**	**7**	**4**	**0.5714**	**0.8000**	**0.6667**
	s1	**7**	**6**	**5**	**0.8333**	**0.7143**	**0.7692**
	s2	**7**	**6**	**5**	**0.8333**	**0.7143**	**0.7692**
